# Modelling GTPase dynamics to understand RhoA-driven cancer cell invasion

**DOI:** 10.1042/BST20160184

**Published:** 2016-12-02

**Authors:** Joseph H.R. Hetmanski, Jean-Marc Schwartz, Patrick T. Caswell

**Affiliations:** Wellcome Trust Centre for Cell-Matrix Research, Faculty of Life Sciences, University of Manchester, Manchester, U.K.

**Keywords:** biological networks, Boolean logic, cell migration, filopodia, RhoA

## Abstract

Metastasis, initially driven by cells migrating and invading through the local environment, leads to most cancer-associated deaths. Cells can use a variety of modes to move *in vitro*, all of which depend on Rho GTPases at some level. While traditionally it was thought that Rac1 activity drives protrusive lamellipodia at the leading edge of a polarised cell while RhoA drives rear retraction, more recent work in 3D microenvironments has revealed a much more complicated picture of GTPase dynamics. In particular, RhoA activity can dominate the leading edge polymerisation of actin to form filopodial actin-spike protrusions that drive more invasive cell migration. We recently described a potential mechanism to abrogate this pro-invasive localised leading edge Rac1 to RhoA switch via manipulation of a negative feedback loop that was revealed by adopting a logical modelling approach. Both challenging dogma and taking a formal, mathematical approach to understanding signalling involved in motility may be vital to harnessing harmful cell migration and preventing metastasis in future research.

## Introduction

An estimated 90% of cancer deaths are caused by secondary tumours [1], the formation of which initially requires malignant cells to invade their local extracellular matrix (ECM) [2]; the understanding of cell migration is therefore of paramount importance to fully combatting the disease. Under physiologically normal conditions in a healthy, fully developed host, most non-haemopoetic and non-immune cells are in a relatively immobile state. Carcinoma cells, however, are driven to transition into mesenchymal, motile phenotypes [3]. While it remains difficult to ascertain the precise strategies adopted by such cells to move and disseminate *in vivo* in humans, an excellent body of experimental findings *in vitro* and *in vivo* demonstrates that cells can migrate individually or collectively in either whole interconnected sheets or more linear chains [4–6]. Individually migrating cells can adopt a variety of different mechanisms to reach their destination: some may protrude their leading edge actin into organised lamellipodia [7] that then adhere to the ECM/substratum in a highly integrin-dependent manner [8] which generates protrusive force [6]; others squeeze through the local environment using membrane blebs and hydrostatic pressure [9]; fibroblasts may even utilise their nucleus as a piston type structure for forward propulsion and movement [10,11]. While there may be many differences between diverse styles of cell motility, it seems clear that all the aforementioned strategies require the role of Rho GTPases at some level [6,12].

## New perspectives on Rho GTPases

**Figure 1.**
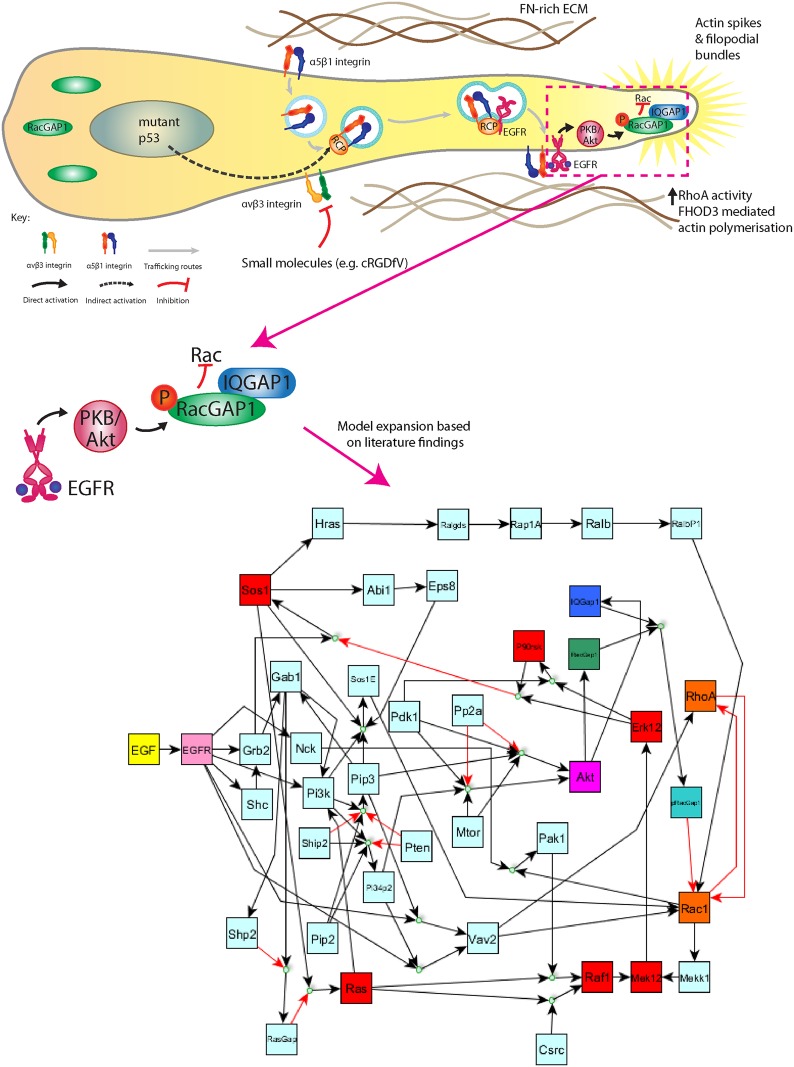
Schematic of known pathway/events leading to invasive RhoA-driven cell migration (top, left) and network representation of Boolean model based on the known priors (bottom, right). Note that the input node EGFR is coloured yellow, the output nodes RhoA and Rac1 are coloured orange, nodes contained in the schematic are coloured correspondingly and all other nodes, implicitly found in the literature, are coloured light grey or red. In the Boolean network, black arrows indicate activation reactions, red arrows indicate inhibition reactions, multiple arrow heads entering directly into a node indicate an OR relation and multiple arrows entering a small circular node indicate an AND relation. The model concerns the immediate transient signalling following EGF binding EGFR after the receptor has been trafficked to the leading edge of a motile cell. Interrogating the model by individual knockout of every node revealed signalling events important for the pro-invasive Rac1 to RhoA switch; in particular, an Sos1 negative feedback loop integral to GTPase dynamics (red nodes).

Rho GTPases, such as RhoA and Rac1, have been recognised as master regulators of cell migration since their discovery 25 years ago [13,14]. These small G proteins play a key role in actin polymerisation via their effect on other proteins such as the Arp2/3 complex [15], Rho-associated kinase (ROCK) [16] and formins [17]. The specific spatiotemporal activity of Rho GTPases is therefore of paramount importance to understanding the dynamics of integrin/adhesion-dependent cell migration. The traditional view, based almost completely on evidence gathered in 2D microenvironments, was that Rac1 dominates at the leading edge of a polarised migrating cell to activate elements, such as the Arp2/3 complex, to promote efficient lamellipodia formation, while RhoA dominates at the rear to mediate actomyosin activity via ROCK and effectively move the rest of the cell body [12]. Importantly, RhoA and Rac1 are mutually antagonistic proteins, whereby it is widely believed that the two GTPases with vastly different signalling roles cannot be active at exactly the same place at the same time in the cell [18,19]. More recent advances in microscopy techniques and studying cells in 3D environments, however, have revealed that Rho GTPase signalling is far more complicated than persistent leading edge Rac1 and rear RhoA activity divided and kept apart by antagonism. While the role of RhoA in cell trailing edge contraction seems well conserved in 3D [20], there has been a suggestion that the efficient formation of protrusions at the leading edge of motile cell requires both Rac1 and RhoA activity in a pseudo-oscillatory manner [21,22]; alternatively, a very tightly regulated band of RhoA activity is required immediately in front of Rac1 activity in a lamellipodium [23]. Moreover, in certain fibronectin-rich ECM conditions, it has been shown that dominant RhoA activity at the leading edge of cells leads to more rapid and random migration in 2D and significantly increased invasion in physiologically relevant 3D microenvironments [24–28]. In cells expressing gain-of-function mutant p53 (associated with increased metastasis), or when αvβ3 integrin is inhibited using cyclic peptides (e.g. cRGDfV) or soluble ligands (e.g. osteopontin), the Rab11 effector Rab-coupling protein (RCP) recruits α5β1 integrin and promotes endocytic recycling and cross-talk between this integrin and epidermal growth factor receptor (EGFR) at the leading edge of invading cells [24]. Upon binding of extracellular EGF to its newly localised receptor, a signalling cascade is potentiated to activate first protein kinase B (PKB, also called Akt), which in turn phosphorylates RacGAP1 [27]. RacGAP1 is a GTPase-activating protein (GAP) specific for Rac1, which binds and hydrolyses guanosine triphosphate (GTP) to guanosine diphosphate (GDP)-bound Rac1, inactivating Rac1 [29]. Following the switching off of the pro-lamellipodial Rac1 activity, which also requires the scaffolding protein IQGAP1 for correct localisation of RacGAP1, the aforementioned antagonism of Rac1 and RhoA leads to increased leading edge RhoA activity [27], presumably in the presence of a RhoA activator or guanine nucleotide exchange factor (GEF), which activates small GTPases by stimulating the release of GDP to allow binding of GTP [29]. RhoA, in turn, activates ROCK, which phosphorylates formin homology 2 domain containing 3 (FHOD3) to release autoinhibition and promote the Arp2/3-independent polymerisation of actin in filopodial spike-like projections at the tips of invasive pseudopodia [28]. This significantly increases the ability of cells to invade fibronectin-rich ECM compared with basal, ‘Rac1-driven’ cells (Figure 1) [24–28].

## Harnessing signalling complexity using simple mathematical logic

The regulation of Rho GTPases by GEFs and GAPs is, while well known, incredibly complicated [29,30]. First, numerous (>20) different GEFs and GAPs are expressed in a variety of different cell types, many with highly conserved functionality [29,30]. Moreover, the question of what regulates the regulators, that is what activates or inhibits the Rho GEFs and GAPs themselves, is far less tractable [31]. Only by fully exploring such signalling can we start to contemplate understanding the complex spatiotemporal dynamics of RhoA and Rac1, which in turn leads to a much better and more predictive knowledge of the essence of cell migration and invasion becoming achievable. Given the complicated, intractable nature of the problem at hand, a more mathematical, logical and formal approach can be of great use [32]. By considering proteins upstream of Rac1 and RhoA as ‘nodes’ in a network, predictions regarding GTPase activity can be made and interrogated following certain simple abstractions. The activity of all nodes (i.e. proteins) contained within the network are binarised into on or off; relations between the nodes are built using the logical operators AND, OR and NOT, and all such relations are assumed to take the same length of time. Others have used a similar approach previously in the context of predicting GTPase activity to highlight the importance of Src and Csk on pro-migratory oscillatory RhoA and Rac1 dynamics [22]. We built a model to determine which perturbations are important for the pro-invasive Rac1 to RhoA switch at the leading edge of cells as recently described [32]. The known linear pathway EGF–EGFR–Akt–RacGAP1, which decreases Rac1 and simultaneously increases RhoA-dominant invasive protrusions [27], is inherently and evidently contained within a much larger and more complicated EGFR signalling network [33]. By studying the existing network pathway maps of EGFR signalling [33–35] and operating an inclusion/exclusion criteria for nodes/relations to include in the network, we built a Boolean logical model which describes the short-term, immediate signalling resulting from EGF–EGFR binding and leading to Rac1/RhoA activity following RCP-α5β1-EGFR trafficking at the leading edge of polarised, motile cells (Figure 1) [32]. This model accurately recreated the pro-invasive Rac1 to RhoA switch observed *in vitro* [27] and highlighted the importance of the Rac1 activator/inhibitor hierarchy *a priori*: the simulations suggested that either Rac1 activity simply gives way to RhoA activity following increased EGF–EGFR binding if RacGAP1 dominates the activity of Rac1 above all activators or, more interestingly, a steady state with RhoA-dominated Rac–RhoA pseudo-oscillations is reached if the Sos1-Eps8-Abi1 Rac1-specific activating complex [36] preferentially acts upon Rac1 over the otherwise dominant RacGAP1. By removing every node in the model and checking the effect on these outputs, we could make testable predictions for possible interventions to abrogate RhoA-driven invasive cell migration. While many of the predictions were well known in the literature, an unexpected negative feedback loop was identified for the Sos1-Eps8-Abi1 complex dominated case: Sos1 activates a signalling cascade whereby Ras [37], Raf [38], MEK1/2 [39], ERK1/2 [40] and p90RSK [41] are activated downstream. ERK1/2 and p90RSK phosphorylate Sos1 to suppress GEF activity however [42], which in Boolean logic terms means that when either are switched ON, Sos1, and thus, the Sos1-Eps8-Abi1 complex is switched OFF. Once Sos1 is switched OFF, all proteins downstream of Sos1 are also eventually switched OFF, including both ERK1/2 and p90RSK, which feeds back to allow Sos1, and its associated Rac1-activating complex to be switched ON again. Breaking this feedback loop at certain points, namely by MEK1/2 removal, was predicted to give rise to sustained Rac1 activity, lamellipodia formation and thus lowered invasive migration in FN-rich matrix. These predictions were apparent both when synchronous (all permissible reactions at any time point occur at said time point) or random asynchronous (one permissible reaction is randomly chosen according to a uniform distribution occurs at each time point) simulations were performed. Indeed, *in vitro* evidence suggested that this prediction was correct: using MEK1/2 inhibitors led to slower, more lamellipodial 2D migration, higher leading edge Rac1 and lower RhoA activity in cells migrating on 3D cell-derived matrix, more lamellipodial actin revealed by super-resolution imaging, and a significantly decreased capacity for cells to invade 3D fibronectin-rich ECM. Abrogating the formation of the Sos1-Eps8-Abi1 complex via Eps8 siRNA, however, rendered cells insensitive to MEK inhibition and returned cells to the RhoA-driven, more invasive filopodial phenotype as predicted by the model [32]. Thus, the model prediction was robustly validated, and a new feedback loop within the EGF signalling network was identified.

## Future directions in cell migration: an integrative 3D approach

Life science research is undergoing a technological and information explosion at present: the tools available in regard to microscopy, mass spectrometry and analysis are of a far higher standard to what was even imaginable during the nascent cell migration and Rho GTPase research conducted 25 years ago. With the wealth of knowledge regarding cell motility growing ever larger, mathematical modelling approaches first help to formally understand and organise known priors, and then in turn make and test predictions regarding gaps in the knowledge. Despite the necessary abstractions, assumptions and simplifications, which naturally come with the Boolean approach, tangible predictions which lead to new biological insights when tested can be made; insights which arguably would never have come to light with a more traditional ‘wet lab’ approach. Using more elegant and complicated mathematical methodologies such as those based on ordinary/partial differential equations or stochastic rule-based models — despite the caveats which come with the difficulty of parameter estimation and the potential of over-determined systems — also have a role to play in move cell migration research forward given their increased quantitative potential. For example, recent modelling approaches have ranged from ordinary differential equations regarding small scale networks of Rho GTPase signalling [43] to more mechanistic approaches with an explicitly spatial macroscopic focus on cell migratory morphologies [44], with a vast array of other methodologies utilised besides [45].

Cancer cell migration research, both *in vitro* and *in silico*, fits within the ultimate aim of preventing fatal metastasis. A potential issue when moved to the scope of the real host human is that of migratory mechanism switching [6]: a therapeutic target may utterly abrogate the signalling leading to one mode adopted by a cell to migrate; however, the said cell may assume a completely different phenotype to metastasise. This issue gives rise to two important factors to consider in cell migration: the need for system-level understanding and the need to challenge dogma and continually look for potential new mechanisms that cells may use to move. A system-level understanding, which is inherently intertwined with adopting a mathematical, network-based approach, could lead to eventually identifying therapeutic combinations to precisely attack multiple key proteins involved in an array of migration signalling pathways, an approach simply not achievable when regarding each pathway as distinct. By challenging the dogma — the view that cells migrate with leading edge Rac1, Arp2/3-driven lamellipodia — more migration mechanisms and signalling pathways have been, and will continue to be, identified. This additional knowledge, when combined and entered into the system-level perspective, may well hold the key for understanding and in turn stopping all manner of cell motility.

## Abbreviations

ECM: extracellular matrix
EGFR: epidermal growth factor receptor
GAP: GTPase-activating protein
GDP: guanosine diphosphate
GEF: guanine nucleotide exchange factor
GTP: guanosine triphosphate
PKB: protein kinase B
RCP: Rab-coupling protein
ROCK: Rho-associated kinase
